# A comparison of micro-CT and histomorphometry for evaluation of osseointegration of PEO-coated titanium implants in a rat model

**DOI:** 10.1038/s41598-017-16465-4

**Published:** 2017-11-24

**Authors:** Tao He, Cong Cao, Zhiguo Xu, Gen Li, Huiliang Cao, Xuanyong Liu, Chao Zhang, Yuqi Dong

**Affiliations:** 1 Department of Orthopedics, Ren Ji Hospital, School of Medicine, Shanghai Jiao Tong University, Shanghai, 200127 P.R. China; 2 State Key Laboratory of High Performance Ceramics and Superfine Microstructure, Shanghai Institute of Ceramics, Chinese Academy of Sciences, Shanghai, 200050 P.R. China

## Abstract

The aim of the present study was to determine the correlation between bone volume density (BV/TV) around a titanium implant determined by micro-computed tomography (micro-CT) and bone area density (BA/TA) measurements obtained using histomorphometry. An intramedullary rat femur implant model was evaluated to compare raw titanium implants with plasma electrolytic oxidation (PEO)-coated titanium implants. Titanium and PEO-treated titanium pins were inserted into rat femurs under general anesthesia. The animals were sacrificed and femurs harvested at 0, 2, 4 and 6 weeks, and subsequently, histomorphometry and micro-CT were performed. BV/TV and BA/TA values were strongly and positively correlated at all time points and locations (with all correlation coefficients being >0.8 and with P < 0.001). BV/TV and BA/TA were significantly higher proximal to the growth plate than distal to the growth plate, with estimated differences of 14.10% (P < 0.001) and 11.95% (P < 0.001), respectively. BV/TV and BA/TA were significantly higher on the PEO-coated surface than on the raw titanium surface, with estimated differences of 3.20% (P = 0.044) and 4.10% (P = 0.018), respectively. Therefore, quantitative micro-CT analysis of BV/TV is correlated with BA/TA determined by histomorphometry when artifacts around titanium implants are minimized by a region of interest modification.

## Introduction

Osseointegration refers to a direct structural and functional connection between ordered, living bone and the surface of a load-carrying implant. Consideration must be given to bone adjacent to the implant and bone that has a structural/functional connection with the implant, as determined by biomechanical evaluation. The success of osseointegration depends on the implant’s surface properties, including the surface morphology, as the roughness affects the implant-bone contact relationship; the chemical composition, as this affects the implant anchorage and the density gradient; the cell factor load, which differs depending on an implant’s source and the manufacturing procedure; and heterotopic bone formation^1–3^. Titanium is the most commonly used implant material, and a number of methods have been used to enhance osseointegration and peri-implant bone formation^3–8^. One such method is surface modification of the implant by plasma electrolytic oxidation (PEO). A PEO-prepared titanium dioxide surface provides a micrometer-scale macro-porous structure that firmly adheres to the titanium substrate^5^. Previous studies have reported that PEO coatings modified by calcium and phosphorus exhibit good bioactivity; however, following modification, the bonding strength of the coating significantly decreases^4–7,9^.

Different techniques have been used to evaluate the response of bone to implants. Among these, histomorphometry remains the most widely used due to its high resolution and good image contrast. However, it is a time-consuming technique that involves complicated procedures, is destructive, collects only two-dimensional (2D) data, and evaluates a limited number of histological sections per implant^10,11^. Therefore, it is important to establish a non-invasive method to evaluate osseointegration.

Micro-computed tomography (micro-CT) is a non-destructive technique that allows precise quantitative three-dimensional (3D) analysis. Many studies have shown the potential of this method for evaluating osseointegration^3,12–22^. However, the accuracy of micro-CT-based quantitative evaluation may be affected by unavoidable metal scatter surrounding the titanium implant due to the implant material^23,24^.

A standard reference volume is required to evaluate the osseointegration of modified implant surfaces and would allow comparability when evaluating the osseointegration of materials with different surface modifications. However, maintaining an identical reference volume across different studies is difficult because the distribution pattern of trabecular bone at the metaphysis varies by millimeters^2^ and the implantation depth is difficult to control. In the distal femoral metaphysis, proximodistal changes of just 1 mm in the implant position may alter the bone volume density (BV/TV) by ~27%^2,25^. Hence, tiny variations in placement depth may significantly affect osseointegration. Previous studies have evaluated the osseointegration of materials with various surface modifications; however, all relevant analyses have focused only on the whole bone volume around the implant, regardless of the influence of the distribution pattern^14,26–28^. Results from different studies are thus difficult to compare.

The aim of the present study was to correlate BV/TV at two levels from the growth plate around a titanium implant using micro-CT. Furthermore, the association between BV/TV as determined by micro-CT and bone area fraction (BA/TA) as determined by histomorphometry in a rat femur implant model was assessed. Additional analyses were performed with untreated titanium rods and those treated with PEO in order to determine the effect of PEO modification on osseointegration.

## Results

### Scanning electron microscopy

**Figure 1:**
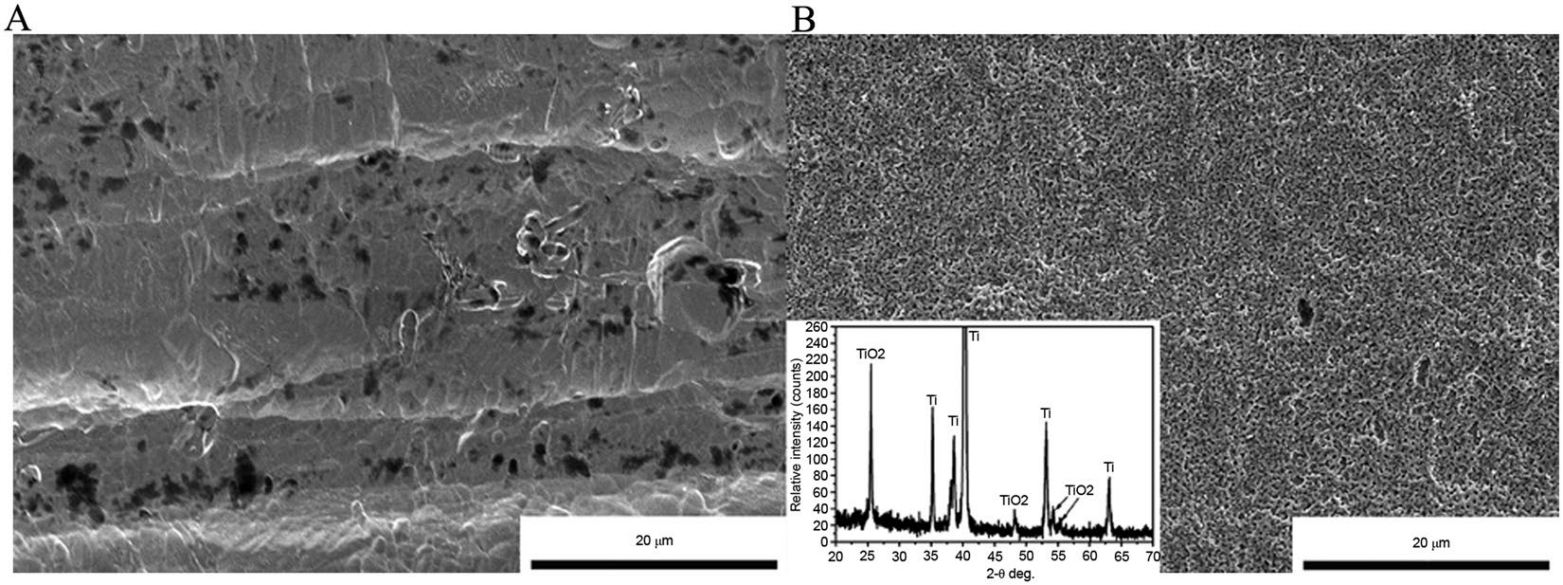
Scanning electron microscopy surface microstructure of the titanium rod (Ф2 mm × 7 mm). (**A**) before and (**B**) after PEO. Following PEO, the surface of the titanium rod became porous. X-ray diffraction patterns (inset in **B**) of the surface indicate that the porous surface layer is composed of PEO. PEO, plasma electrolytic oxidation.

Scanning electron microscopy indicated that the raw titanium implants had a relatively smooth surface (Fig. 1A). After PEO, the implant surface exhibited a macroporous structure (Fig. 1B). In addition, the thickness of the PEO coating was ~10 μm. Due to the nature of the PEO process, the pores in the coating were not interconnected; therefore, the porosity of the PEO surface could not be tested.

### Correlation between histomorphometry and micro-CT

**Figure 2:**
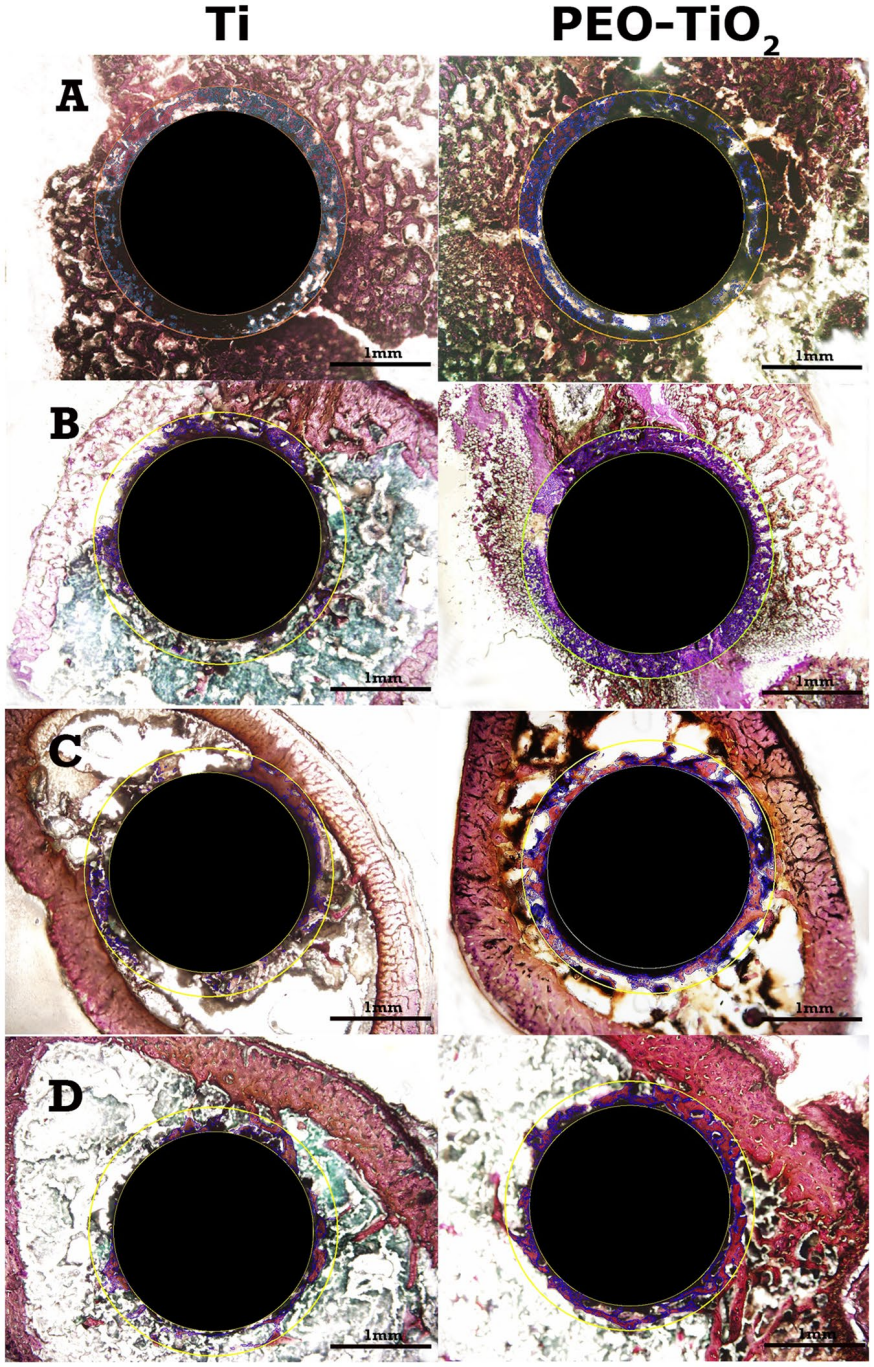
Representative histomorphometric images of tissue sections associated with titanium (Ti, left) and PEO-modified (PEO-TiO_2_, right) implants (**A**) 0 weeks, (**B**) 2 weeks, (**C**) 4 weeks and (**D**) 6 weeks after implantation into rat distal femurs. PEO, plasma electrolytic oxidation.

**Figure 3:**
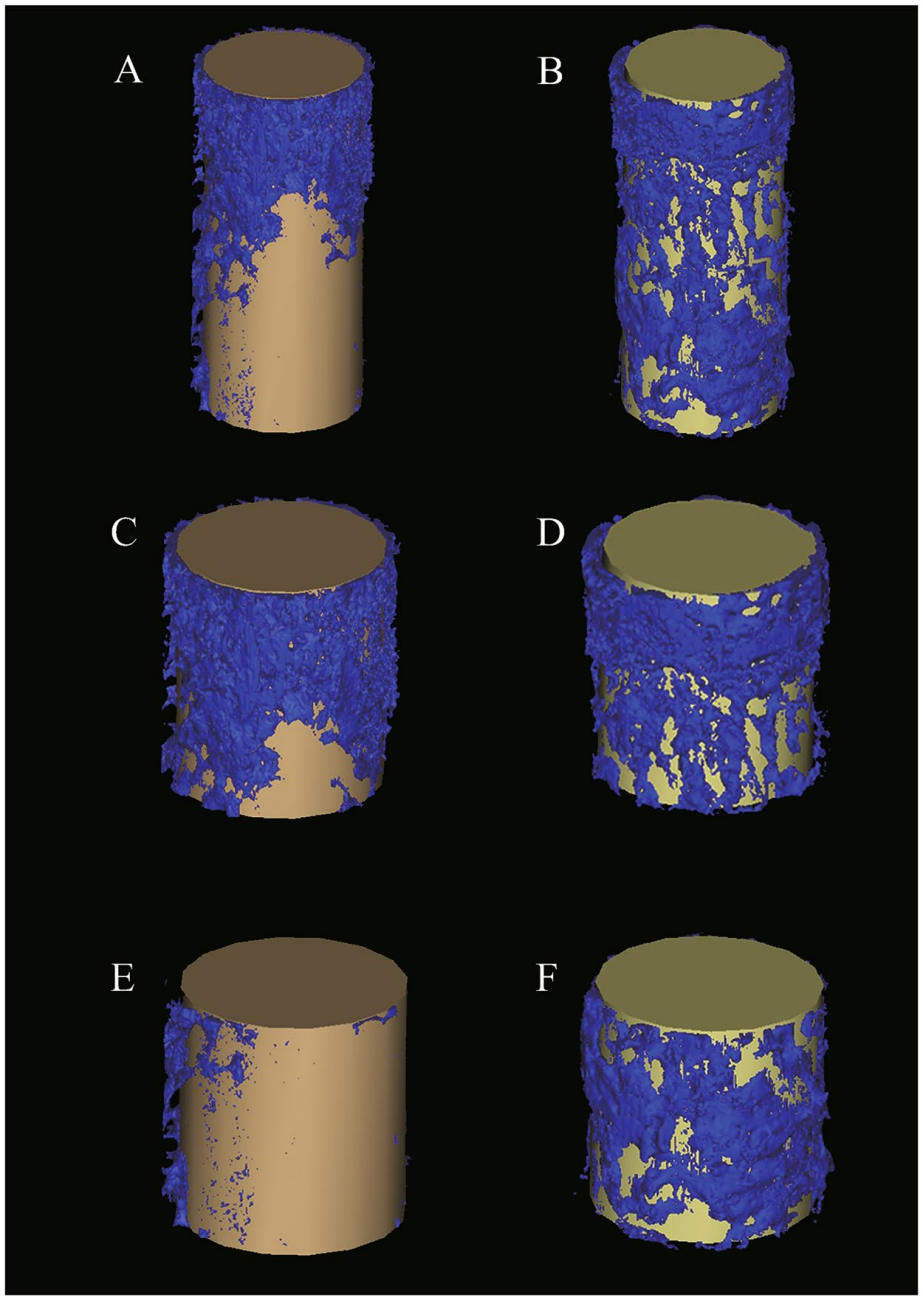
Representative 3D representations of micro-computed tomography images of implant and bone volume around implants at 4 weeks. Micro-computed tomography images of (**A**) total titanium implants, (**B**) total PEO-coated implants, (**C**) proximal titanium implants, (**D**) proximal PEO-coated implants, (**E**) distal titanium implants, and (**F**) distal PEO-coated implants. PEO, plasma electrolytic oxidation.

**Figure 4:**
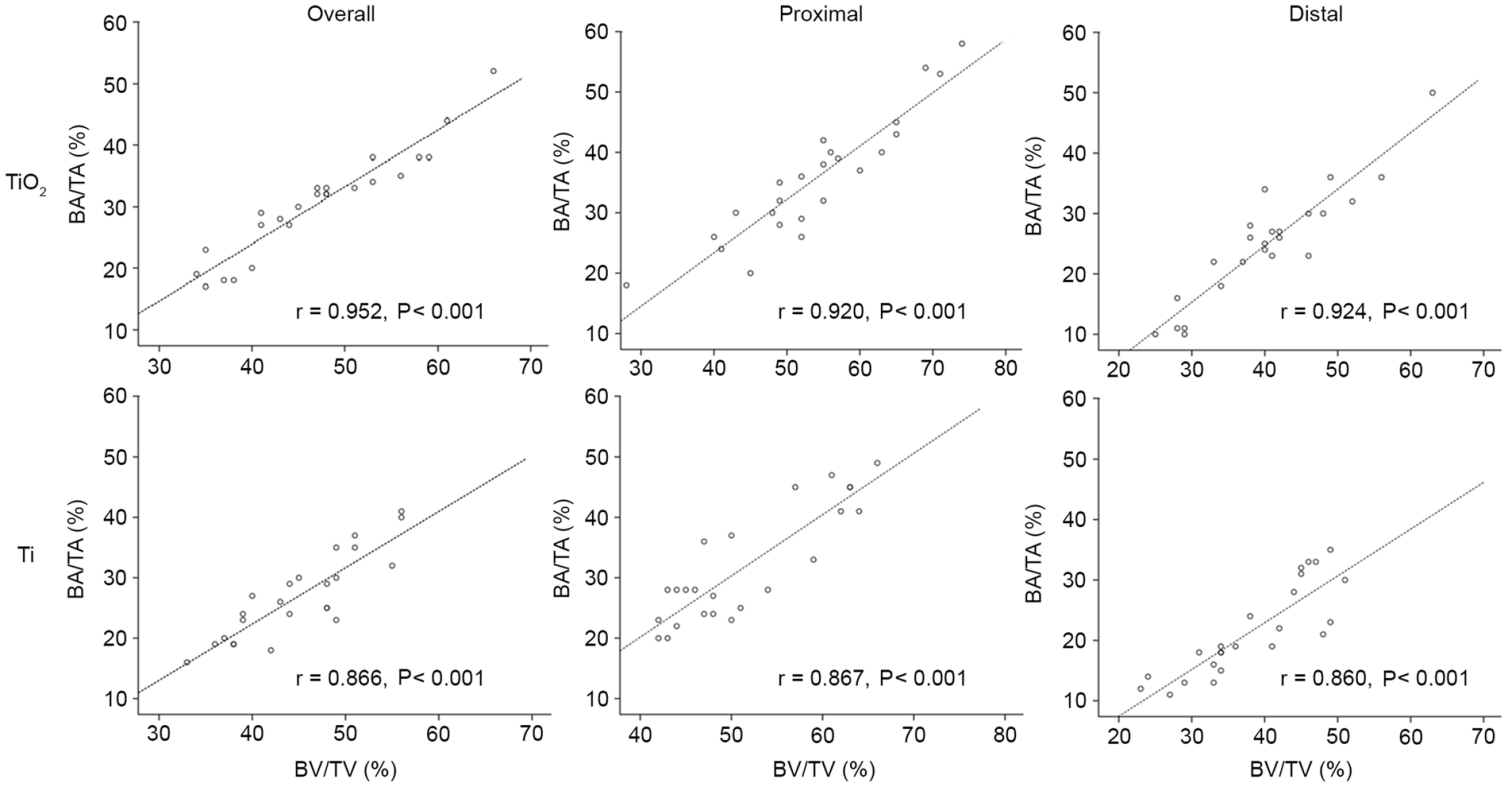
Correlation between BA/TA and BV/TV. BA/TA and BV/TV in overall, proximal and distal sites of TiO_2_ implants exhibited a strong positive correlation (P < 0.001). BA/TA and BV/TV in overall, proximal and distal sites of Ti implants also exhibited a strong positive correlation (P < 0.001). BA/TA, bone area density; BV/TV, bone volume density. TiO_2_, plasma electrolytic oxidation-coated implants; Ti, titanium implants.

**Figure 5:**
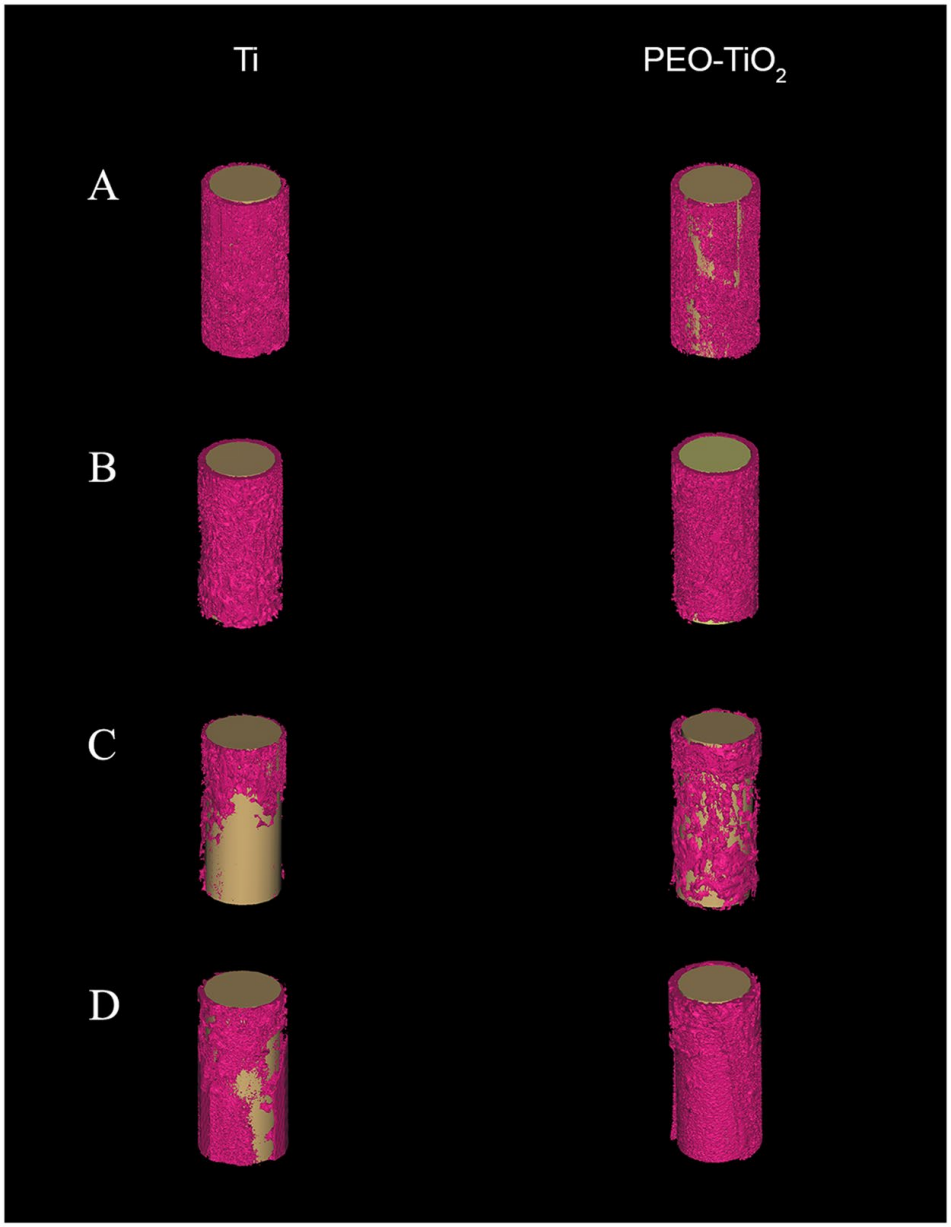
Representative 3D representations of micro-computed tomography images of titanium (Ti, left) and PEO-modified (PEO, right) implants after (**A**) 0, (**B**) 2, (**C**) 4 and (**D**) 6 weeks of implantation into rat distal femurs. PEO, plasma electrolytic oxidation.

Representative histomorphometry and micro-CT images are depicted in Figs 2 and 3, respectively. Curve-fitting analysis of the BV/TV values obtained from micro-CT and BA/TA values obtained from histomorphometry revealed a strong positive correlation regardless of time point or location (all correlation coefficients >0.8 with P < 0.001; Fig. 4). Proximal and distal micro-CT images of PEO-coated and raw titanium implants at the 0-, 2-, 4- and 6-week time points to observe changes and the difference between the two implants over time are presented in Fig. 5. These data in the figure indicate that the bone volume around the PEO-coated implants at 4 and 6 weeks was greater than that around the raw titanium implants, particularly at the distal portion of the implants.

### Effects of time, surface type, and location on osseointegration

BV/TV and BA/TA were significantly higher in the region proximal to the growth plate compared with the distal region, with estimated differences of 14.69% (P < 0.001) and 11.85% (P < 0.001), respectively. This effect was present irrespective of surface coating.

BV/TV at 2 and 4 weeks was significantly higher than that at week 0, with estimated differences of 14.51% (P < 0.001) and 4.54% (P = 0.004), respectively. Similar results were observed regarding BA/TA; BA/TA at 2 and 4 weeks was significantly higher than that at week 0, with estimated differences of 14.85% (P < 0.001) and 5.00% (P = 0.005), respectively. Furthermore, BV/TV and BA/TA were significantly higher in the PEO surface compared with the Ti surface, with estimated differences of 2.70% (P = 0.022) and 4.14% (P = 0.002), respectively (Table 1).

**Table 1:** Effects of time, surface type and location on osseointegration.

		BV/TV (%)		BA/TA (%)	
		Effects (95% CI)	P-value	Effects (95% CI)	P-value
Time	0 weeks	Reference		Reference	
	2 weeks	14.51 (11.51, 17.51)	<0.001^a^	14.85 (10.85, 18.85)	<0.001^a^
	4 weeks	4.54 (1.56, 7.51)	0.004^a^	5.00 (1.58, 8.41)	0.005^a^
	6 weeks	0.53 (−3.18, 4.24)	0.774	1.58 (−2.52, 5.68)	0.439
Surface	PEO	2.70 (0.40, 5.00)	0.022^a^	4.14 (1.61, 6.67)	0.002^a^
	Ti	Reference		Reference	
Location	Distal	−14.69 (−17.12, −12.27)	<0.001^a^	−11.85 (−14.73, −8.97)	<0.001^a^
	Proximal	Reference		Reference	

^a^P < 0.05 indicates that the fixed effects were statistically significant in the linear mixed model. BV/TV, bone volume density; BA/TA; bone area density; CI, confidence interval; PEO, plasma electrolytic oxidation; Ti, titanium.

### Osseointegration of distal and proximal regions

Differences in BV/TV between distal and proximal regions of PEO-coated implants were significant at 0 weeks (34.50 vs. 48.00%, P = 0.003), 2 weeks (52.33 vs. 65.50%, P = 0.020) and 4 weeks (39.17 vs. 58.17%, P = 0.001; Table 2). Differences in BV/TV between distal and proximal regions of raw titanium implants were significant at 2 weeks (46.00 vs. 59.83%, P = 0.008), 4 weeks (35.17 vs. 51.5%, P = 0.021) and 6 weeks (34.83 vs. 47.83%, P = 0.034).

**Table 2:** Comparison of osseointegration proximal and distal to the growth plate.

		BV/TV (%)			BA/TA (%)		
		Distal	Proximal	P-value	Distal	Proximal	P-value
PEO	0 weeks	34.50 (4.85)	48.00 (5.29)	0.003^a^	21.83 (9.60)	29.50 (2.95)	0.057
	2 weeks	52.33 (6.28)	65.50 (6.95)	0.020^a^	34.50 (8.98)	47.00 (8.99)	0.062
	4 weeks	39.17 (3.06)	58.17 (5.53)	0.001^a^	23.33 (3.20)	40.50 (3.39)	0.001^a^
	6 weeks	34.83 (8.86)	43.83 (8.80)	0.173	19.83 (8.61)	25.50 (6.06)	0.190
Ti	0 weeks	36.83 (11.74)	47.33 (2.80)	0.068	21.17 (9.62)	26.50 (5.89)	0.057
	2 weeks	46.00 (4.60)^b^	59.83 (5.38)	0.008^a^	28.50 (5.32)	40.33 (9.27)^b^	0.011^a^
	4 weeks	35.17 (5.56)	51.50 (8.76)	0.021^a^	16.83 (3.76)^b^	32.50 (9.31)	0.025^a^
	6 weeks	34.83 (5.56)	47.83 (8.18)	0.034^a^	19.67 (5.99)	28.50 (8.34)	0.122

The data are presented as the mean ± standard deviation. ^a^P < 0.05 indicates a significant difference between distal and proximal regions. ^b^P < 0.05 indicates a significant difference in osseointegration between PEO and Ti at a given time and site. BV/TV, bone volume density; BA/TA; bone area density; PEO, plasma electrolytic oxidation; Ti, titanium.

Differences in BA/TA were significant between distal and proximal regions of PEO-coated implants at 4 weeks (23.33 vs. 40.50%, P = 0.001) and of raw titanium implants at 2 weeks (28.50 vs. 40.33%, P = 0.011) and 4 weeks (16.83 vs. 32.5%, P = 0.025).

Significant time-specific and site-specific differences between the PEO and raw titanium groups were observed for distal BV/TV at 2 weeks (52.33 vs. 46.00%, P = 0.027), proximal BA/TA at 2 weeks (47.00 vs. 40.33%, P = 0.041) and distal BA/TA at 4 weeks (23.33 vs. 16.83%, P = 0.008; Table 2).

## Discussion

The results of the present study indicated that BV/TV around a titanium implant as determined by micro-CT correlated with BA/TA obtained from histomorphometry. Furthermore, BV/TV and BA/TA were greater in the area proximal to the growth plate. Both BV/TV and BA/TA were significantly higher in the PEO-treated surface than in the raw titanium surface.

Modified titanium surfaces demonstrate better bioactivity compared with pure titanium surfaces. However, the effect of osseointegration of different modified surfaces is difficult to compare because there is not a unified reference volume. To evaluate osseointegration, histomorphometric analysis focuses on limited sections from the whole bone volume around the implant^4^. Although this evaluation produces quantitative results, the 2D data do not reflect the actual 3D structure of bone. Previous studies have evaluated the total bone volume around an implant using micro-CT imaging to obtain a 3D image of the structure. Micro-CT offers more comprehensive and accurate information than traditional methods and has produced encouraging results^12,14,28–30^. Vandeweghe *et al*.^31^ determined that BA and bone-implant contact (BIC) data obtained from histomorphometry and micro-CT, respectively, correlated well, although the histomorphometry values were slightly higher than those obtained with micro-CT.

However, micro-CT has several drawbacks. First, artifacts around the metal implant decrease the accuracy of micro-CT results^23,24^. Schouten *et al*.^27^ found that artifacts in the inner area of the ROI resulted in abnormally high bone volume values during micro-CT analysis compared with those obtained via histomorphometry. However, in the middle and outer areas of the ROI, the osseointegration results obtained via the two methods correlated well. Second, the implantation depth significantly affects osseointegration because the distribution of trabecular bone varies with distance from the metaphysis. To avoid these problems and minimize the interference of artifacts, the ROI around the implant was optimized, and the BV/TV and BA/TA were measured at two distances from the growth plate; the results revealed that when the ROI is modified, a good correlation between micro-CT and histomorphometry is achieved. Nevertheless, a recent study demonstrated that osseointegration is compromised by radiation in a dose-dependent manner^32^. Therefore, micro-CT scanning should reduce the radiation dosage as much as possible without sacrificing the quality of the images.

In the present study, quantitative micro-CT analysis correlated very well with the histomorphometric observations, although the percentages obtained via micro-CT imaging were ~20% higher than those obtained via histomorphometry, in contrast to the results of Vandeweghe *et al*.^31^ This discrepancy may be explained by the manual selection of the threshold for trabecular bone during micro-CT analysis in the current study. The results of the present study also indicated that artifacts were still present in the ROI, and the threshold of these artifacts was similar to that of trabecular bone. Thus, the values obtained by micro-CT were higher than those obtained via histomorphometry.

Chen *et al*.^33^ reported that bone remodeling in an immature rabbit model reached a peak at 6 weeks, as woven bone and new trabeculae formation decreased after 5 weeks, leading to a decrease in the bone volume around the implant at 6 weeks. Moreover, Irish *et al*.^34^ reported that bone remodeling began at 8 weeks in a mature male rat model. In the present study, to obtain a greater trabecular response to the implants, we chose immature rats as the animal model^35^. It should be noted that at 6 weeks, BV/TV and BA/TA appeared to be decreasing. Because immature rats were used, the remodeling process was likely faster than it would have been in mature rats; therefore, this may explain the decrease in BV/TV and BA/TA observed at 6 weeks.

It has been demonstrated that PEO coatings exhibit bioactive properties when combined with certain materials, including tricalcium phosphate^5^. In the present study, increases in BV/TV at early stages of osseointegration were observed with the PEO-coated implants. Nanometer-scale porous structures are known to induce osseointegration^4,8,9^, which may explain why the PEO coatings exhibited good bioactivity *in vivo* without any other modifications. Moreover, since the ceramic coating is stable and difficult to separate from the titanium substrate, it offers excellent stability at the bone-implant interface^36^.

BIC, BV/TV, bone mineral density, BA/TA, mean trabecular thickness, mean trabecular number and mean trabecular separation have been used to quantify the response at the bone-implant interface^30,37,38^. Studies have shown that bone contact exerts the most influence with respect to the stability of the bone-implant interface. Diefenbeck *et al*.^4^ reported that BIC correlates well with biomechanical testing data and can therefore be considered a more ideal parameter for evaluating implant anchorage than BA. Additionally, Gao *et al*.^39^ identified a similar correlation between BIC and shear strength but not between BA and shear strength. However, BIC is poorly visualized on micro-CT images due to artifacts from the metal implant. Thus, micro-CT is not suitable for evaluating BIC. For this reason, BV/TV was selected to evaluate osseointegration in the current study. Although BV/TV was correlated with BA/TA in the present study, a number of studies have indicated that BV/TV is not correlated with biomechanical data^40–42^. When studying osseointegration, it must be kept in mind that there is a difference between bones being present adjacent to the implant and a structural and functional connection between bone and the implant as determined by biomechanical evaluation. The present study did not perform a biomechanical evaluation, and thus, it cannot be stated with certainty that structural/functional osseointegration occurred. Therefore, it cannot be precisely concluded that BV/TV determined by micro-CT is effective at evaluating osseointegration. However, in the present study, the ROIs were limited to a 190-μm circular band stretching from 60 to 250 μm from the implant surface and outward, indicating close contact between the bone and the implant and suggesting osseointegration. In another experiment using a different set of samples, push out testing was performed, and it was determined that BV/TV was positively correlated with the mechanical force required to remove the implant^43^.

The present study had several limitations, in addition to the aforementioned lack of mechanical data. A histological analysis of osseointegration was not performed. Although the accuracy of micro-CT-based quantitative analysis may be affected by artifacts due to the implant materials, the present study only evaluated two titanium implants *in vivo*. Therefore, future studies that evaluate different implant materials are required to demonstrate that micro-CT results consistently correlate with those of histomorphometric analysis. In addition, the difference in BV/TV and BA/TA between the PEO-treated group and the Ti group at week 6 was minimal when considering time and location, likely due to the small sample size and the nature of osseointegration, which occurred very early on in the experiment. Furthermore, the current study did not include a sham-operated group without implants. Lastly, given the limited sample and effect size (i.e., the mean difference between surface types), post hoc calculations of power for the comparison of differences between surface types at given time points and locations ranged between 0.05 and 0.49, apart from the comparison of the distal site at week 4 (power = 0.94).

Quantitative micro-CT analysis of BV/TV correlates with BA/TA determined by histomorphometry when artifacts around titanium implants are minimized by ROI modification. BV/TV and BA/TA were greater in the region proximal to the growth plate and were enhanced by PEO treatment of titanium implants at the very early phase after implantation, which lessened the bone loss from weeks 2 to 6. However, further mechanical testing experiments are necessary to determine whether micro-CT may be used for evaluation of osseointegration of titanium implants.

## Materials and Methods

### Animal model

All experiments were approved by the Animal Care Committee of Shanghai (Reg. no. SCXK2004-0001, Shanghai, China). A total of 24 8-week-old male Sprague-Dawley rats (weight, 232.11 ± 20.33 g) were purchased from Shanghai Sippr-BK Laboratory Animal Co., Ltd. (Shanghai, China) and were given free access to water and standard rat food (protein 226 g/kg, fat 50 g/kg and metabolizable energy 3,030 kcal/kg). Rats were kept under a 12-h light/dark cycle. Immature rats were selected, as osseointegration is more easily observed in immature rather than mature animals. Moreover, the environment was maintained at a temperature between 20 and 24 °C and a relative humidity between 40 and 70%. All experiments were performed and animals treated in accordance with the Guide for the Care and Use of Laboratory Animals^44^.

### Titanium rods

In total, 48 commercially available pure titanium (99.9%) rods (Xi’an Saite Metal Materials Development Co., Ltd., Xi’an, China) measuring 2 × 7 mm were used in the present study. Among the 48 rods, 24 received a PEO coating, and 24 were used as controls without any coating. All rods were washed with acetone, ethyl alcohol and distilled water in an ultrasonic bath (40 kHz; B8510; Branson Ultrasonics, Danbury, CT, USA) prior to use.

### PEO surface preparation

PEO of the rods was performed in an electrolyte solution (Sinopharm Chemical Reagent Co., Ltd., Shanghai, China) containing 0.05 mol/l glycerophosphate disodium salt pentahydrate (C_3_H_7_Na_2_O_6_P × 5H_2_O, chemically pure reagent) and 0.5 mol/l sodium silicate (Na_2_SiO_3_, analytically pure reagent) using a commercially available device (MAO-30; Pulsetech Products Corporation, Southlake, TX, USA). The applied current density, frequency, duty cycle and duration time were 30 A/dm2, 600 Hz ± 30% and 10 min. In addition, titanium rods were used as the anode, and a spiral steel pipe was used as the cathode; a dwelling water pipe was also used to maintain the temperature of the electrolyte solution <30 °C. A magnetic stirrer (600 rpm; Shanghai Mei Yingpu Instrument Manufacturing Co., Ltd., Shanghai, China) was used to ensure the uniformity of the components and temperature of the electrolytes. Following PEO treatment, the samples were washed with deionized water and air-dried for 1 h. The PEO coating did not appear to increase the diameter of the implants, as the major component of the coating was titanium oxide. The thickness of the coating used in the present study was ~10 μm.

### Inspection of implant surfaces via scanning electron microscopy (SEM)

Sample PEO and raw titanium implants that were not used for surgery were examined with SEM (Zeiss Supra 55VP; Zeiss AG, Oberkochen, Germany). The implants were gold coated (108 Auto Sputter Coater; Ted Pella, Inc., Redding, CA, USA) before SEM observation.

### Surgical procedure

Prior to implantation, the implants were sterilized at 134 °C and a pressure of 2.16 bar for 35 min. Surgery was performed on rats under general anesthesia with intraperitoneal injection of 0.15 mg/kg dexmedetomidine (Pfizer GmbH, Berlin, Germany), 2.0 mg/kg midazolam (Ratiopharm, Ulm, Germany) and 0.005 mg/kg fentanyl (Janssen-Cilag GmbH, Neuss, Germany). Animals were prepared for surgery by shaving both hind legs and disinfecting them with 75% alcohol. The entire surgical procedure was performed in sterile conditions using sterile drapes and sheets, and both hind legs were draped with sterile gauze. A 1-cm longitudinal medial incision was made to expose the knee joint in both hind limbs, and a pilot hole was marked at the intercondylar eminence. A custom-made awl with a tip size of 2.0 mm in diameter and 10 mm in length was gradually rotated on the bone to create a channel from the distal femoral epiphysis into the medullary canal. This channel measured 2 mm in diameter and 10 mm in length. The implants were then inserted via the channel and positioned 2 mm from the articular cartilage. In each of the 24 rats, a raw titanium rod was placed in the right hind leg and a PEO-coated rod in the left hind leg (paired design).

**Figure 6:**
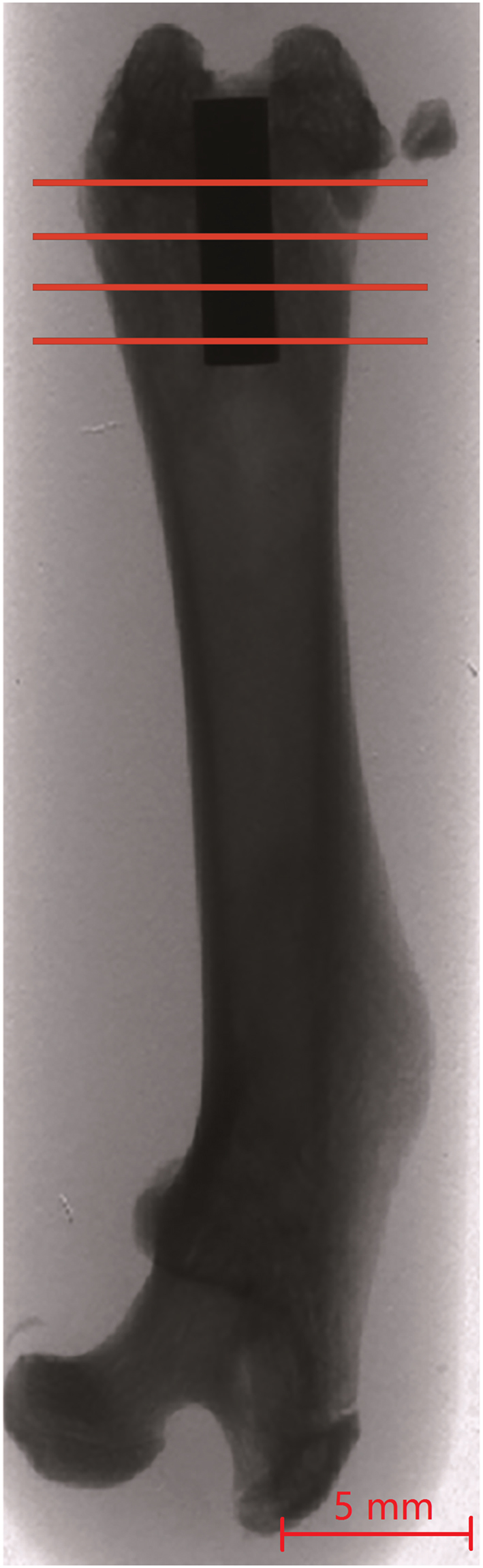
Radiographs indicating the position of the implant within a rat femur. The lines illustrate the four cutting levels. Scale bar, 5 mm.

The soft tissue was irrigated with sterile saline, and the fascia and skin incisions were closed via the single-knot technique. In addition, prophylactic antibiotic (6 mg/kg oxytetracycline; Pfizer GmbH) and analgesic (0.05 mg/kg buprenorphine; Bayer AG, Leverkusen, Germany) were administered once, intramuscularly, at the time of surgery. The implant position within each rat femur was confirmed by X-ray (Fig. 6).

### Explant procedure

At each time point (0, 2, 4 and 6 weeks following surgery), 6 rats were sacrificed, and femurs were harvested and cleared of all soft tissue. All femurs were kept in 75% alcohol at 4 °C prior to micro-CT scanning and histological examination. During CT scanning, the specimens were kept in 75% alcohol to prevent them from drying.

### Micro-CT analysis of bone volume (BV/TV)

Micro-CT was performed prior to histomorphometric analysis to identify the exact position of the growth plate. Scanning was performed with a high-resolution micro-CT scanner (eXplore Locus SP, GE Healthcare Bio-Sciences, Pittsburgh, PA, USA), and specimens were placed vertically into a sample holder and scanned. Scanning was performed at 90 kV and 80 μA. A copper filter (GE Healthcare Bio-Sciences) was applied to optimize the contrast, and the exposure time was 1,700 msec. The nominal resolution was 16 μm, and 3D image reconstruction was performed with MicroView version 2.0 software (GE Healthcare Life Sciences). Quantitative data from images were obtained with Mimics software (version 10.01; Materialise, Leuven, Belgium).

Regions of interest (ROIs) in the proximal portion of each femur started from the proximal border of the growth plate. Two ROIs of 2.5 mm each (0.0–2.5 and 2.5–5.0 mm from the growth plate border) were selected, and the average of the two was used for the analysis. Distal ROIs starting from the distal border of the growth plate were then selected in the same way as the proximal ROIs. All images were individually evaluated, and the ROI mask was applied to each image. A threshold of 1,000–2,500 Hounsfield units in the ROI was used to identify bone. ROIs were limited to a 190-μm circular band stretching 60–250 μm from the implant surface and outward. This circular band was selected to eliminate the effects of artifacts from the implant surface, as a distance of 60 μm from the implant surface has been demonstrated to be sufficiently far from the metal to minimize artifacts^45^. A value of 250 μm was selected for the outer circumference of the circular band because it is 25% of the radius of the implant, a commonly used distance^4^. The 3D image analysis using micro-CT measures true 3D thickness, which is model independent^37^.

### Histomorphometric analysis of BA/TA

As previously described^4^, distal femurs with *in situ* implants were fixed in 5% neutral-buffered formalin for 10 days at room temperature and dehydrated with increasing concentrations of alcohol. Specimens were then impregnated with a 1:1 mixture of absolute alcohol and Technovit 7200 VLC (Bio Optica Milano SpA, Milan, Italy) and then embedded in pure Technovit 7200 VLC without decalcification.

Since micro-CT allows for easy identification of the growth plate, we performed the micro-CT scan before each histomorphometric analysis in order to find the exact position of the growth plate. We then sliced each histological specimen at the same layer as that used for the micro-CT analysis. This allowed accurate comparison of the two methods. Cross-sections (200 μm) transverse to the longitudinal axis of the femur were made using an EXAKT 300 diamond band saw (Exakt Technologies, Inc., Oklahoma City, OK, USA) with a blade width of 130 μm. Cross-sections were cut at the same layers used for micro-CT analysis (with the same sub-ROIs); thus, four sections, two proximal and two distal to the growth plate, were obtained. In addition, the 200-μm sections were ground to a thickness of 50 μm using an EXAKT 400CS grinding system (Exakt Technologies, Inc.). As the tissue loss due to the blade width was 130 μm and the 200-μm sections were ground to a thickness of 50 μm, the total loss per blade cut was 130 + 150 = 280 μm. Sections were stained using the Van Gieson method^46^ and visualized using a light microscope.

**Figure 7:**
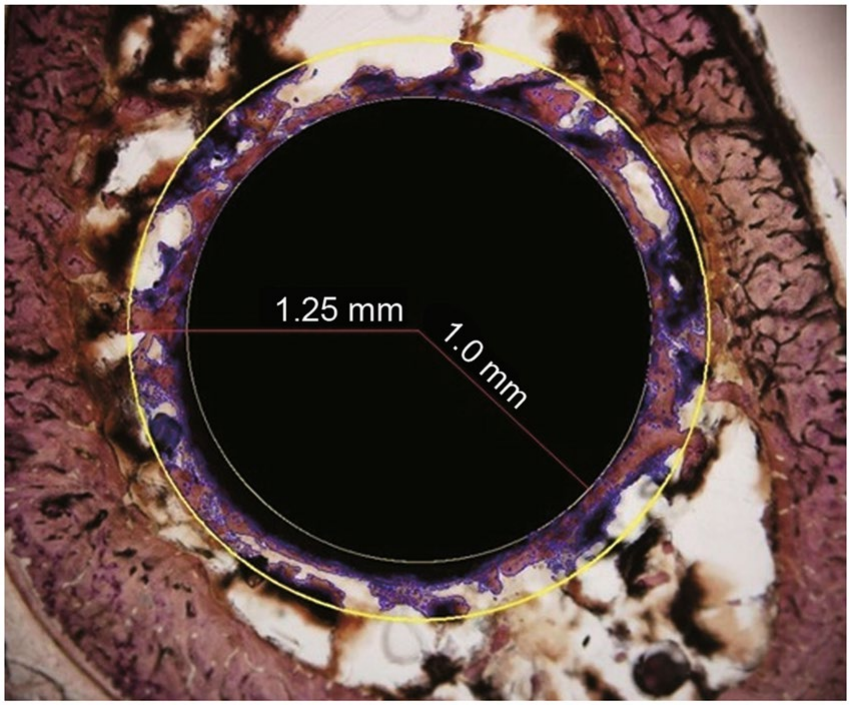
Histomorphometric analysis of the bone area density. Within a circular band around the implant that was 250 μm (0.25 mm) thick, the red-dyed bone was marked, and the bone area (using only the red-dyed areas) was calculated. BA/TA (%) = bone area in the band/total area of the band. A representative image of a PEO implant at 4 weeks is presented (Van-Gieson stain; magnification, ×4).

The percentage bone area was determined using a semi-automated digitized image analyzer system consisting of an Axioplan II stereomicroscope, a computer-coupled AxioCam, HRC and Axivision 4.7 software (Carl Zeiss AG). Within a circular band around the implant that was 250 μm thick, the red-dyed bone was marked, and the bone area (only the red-dyed area) was calculated. The results of two sections from the same area were averaged. The ROI was defined as a circular band 250 μm from the implant surface, and the area with bone was estimated and BA/TA calculated using the formula BA/TA (%) = bone area in the band/total area of the band (Fig. 7).

### Statistical analysis

BV/TV and BA/TA at various time points (0, 2, 4 and 6 weeks) and locations (distal/proximal) were presented as the mean ± standard deviation. Pearson correlation coefficients were calculated to evaluate the correlation between BV/TV and BA/TA. Linear mixed models were also performed to evaluate the effects of the four time points (0, 2, 4 and 6 weeks), the two types of surfaces (PEO and Ti) and the two locations (distal and proximal to the growth plate) on BV/TV and BA/TA. A paired t-test was performed to compare the differences in BV/TV and BA/TA between the distal and proximal regions, with stratifications for the type of surface and time point. A time point of 0 weeks was used as a reference for determining the changes in BV/TV and BA/TA. Moreover, all statistical assessments were two-sided, and P < 0.05 was used to indicate a statistically significant difference. Statistical analyses were performed using SPSS software version 15.0 (SPSS, Inc., Chicago, IL, USA).

### Data availability

The datasets generated and/or analyzed during the current study are available from the corresponding author upon reasonable request.
